# Inflammatory biomarkers and subclinical carotid atherosclerosis in HIV-infected and HIV-uninfected men in the Multicenter AIDS Cohort Study

**DOI:** 10.1371/journal.pone.0214735

**Published:** 2019-04-04

**Authors:** Vinita Subramanya, Heather S. McKay, Rebeccah M. Brusca, Frank J. Palella, Lawrence A. Kingsley, Mallory D. Witt, Howard N. Hodis, Russell P. Tracy, Wendy S. Post, Sabina A. Haberlen

**Affiliations:** 1 Division of Cardiology, Johns Hopkins School of Medicine, Baltimore, Maryland, United States of America; 2 Department of Epidemiology, Johns Hopkins Bloomberg School of Public Health, Baltimore, Maryland, United States of America; 3 Department of Medicine, Johns Hopkins School of Medicine, Baltimore, Maryland, United States of America; 4 Division of Infectious Diseases, Northwestern University, Feinberg School of Medicine, Chicago, Illinois, United States of America; 5 Department of Epidemiology, University of Pittsburgh School of Public Health, Pittsburgh, Pennsylvania, United States of America; 6 Department of Medicine, Los Angeles Biomedical Research Institute at Harbor-University of California Los Angeles, Torrance, California, United States of America; 7 Atherosclerosis Research Unit, Keck School of Medicine at University of Southern California, Los Angeles, California, United States of America; 8 Departments of Pathology & Laboratory Medicine, and Biochemistry, University of Vermont, Larner College of Medicine, Colchester, Vermont, United States of America; University of Pittsburgh Centre for Vaccine Research, UNITED STATES

## Abstract

**Background:**

HIV-infected persons have an increased risk of atherosclerosis relative to uninfected individuals. Inflammatory processes may contribute to this risk. We evaluated the associations of 10 biomarkers of systemic inflammation (CRP, IL-6, sTNF-αR1 and 2), monocyte activation (CCL2, sCD163, sCD14), coagulation (fibrinogen, D-dimer), and endothelial dysfunction (ICAM-1) with subclinical carotid atherosclerosis among participants in the Multicenter AIDS Cohort Study (MACS).

**Methods:**

Carotid plaque and intima media thickness (IMT) in the common carotid (CCA-IMT) and bifurcation region were assessed by B mode ultrasound among 452 HIV-infected and 276 HIV-uninfected men from 2010–2013. Associations between levels of each biomarker and presence of focal plaque and IMT were assessed by logistic and linear regression models, adjusting for demographics, risk behaviors, traditional cardiovascular disease (CVD) risk factors, and HIV disease characteristics.

**Results:**

Compared to HIV-uninfected men, HIV-infected men had significantly higher levels of 8 of the 10 biomarkers. Overall, men with sCD163, CCL2, IL-6, and CRP levels in the highest quintile had approximately 2 times the odds of carotid plaque relative to those with levels in the lowest quintile, independent of demographic and CVD risk factors. Fibrinogen levels were positively associated with CCA-IMT while ICAM-1, CCL2, and sTNF-αR1 levels were positively associated with bifurcation-IMT. Among HIV-uninfected men, higher levels of sTNF-αR2 were positively associated with CCA-IMT, fibrinogen with bifurcation-IMT and carotid plaque, and ICAM-1 with carotid plaque.

**Conclusion:**

In addition to greater levels of systemic inflammation, heightened monocyte activation (sCD163, CCL2) may contribute to the burden of atherosclerosis among HIV-infected persons.

## Introduction

HIV-infected individuals have a greater risk of subclinical atherosclerosis and cardiovascular disease (CVD) events, even among those with suppressed HIV RNA levels [1–3], compared to HIV-uninfected persons [4, 5]. Mechanisms underlying this increased HIV-associated risk may include HIV viremia, immune activation, metabolic side effects of antiretroviral therapy, and a higher prevalence of traditional CVD risk factors among HIV-infected persons [6–8]. Dysregulation of the immune system, persistent inflammation, and endothelial dysfunction, possibly as a consequence of ongoing residual low-level viral replication, may play a central role in excess CVD risk among HIV-infected persons [2, 5, 9–12].

Although systematic reviews demonstrate mixed results regarding the associations between levels of specific biomarkers and surrogate markers of cardiovascular disease among HIV-infected persons [13, 14], studies have reported associations between levels of CRP, IL-6, TNF alpha receptors 1 and 2 (sTNF-αR1 and 2), sCD14, sCD163, CCL2, and D-dimer with subclinical or incident CVD [5, 11, 14–18]. However, many studies had small sample sizes, lacked an HIV-uninfected comparison group, or did not adequately adjust for confounding factors. Moreover, comparisons across studies are complicated by the differences in subclinical atherosclerosis measurement, including carotid intima-media thickness (IMT), carotid and coronary plaque, and coronary artery calcium.

In previous reports involving HIV-infected and uninfected men in the Multicenter AIDS Cohort Study (MACS), prevalent subclinical coronary atherosclerosis was positively associated with elevated levels of monocyte activation biomarkers—sCD14, sCD163, CCL2 (previously MCP-1)—[18] and other biomarkers of inflammation (IL-6, ICAM-1, and sTNF-αR1 and 2) [19], independent of traditional cardiovascular risk factors. Building on this prior work, the aim of the current study was to evaluate the relationship between levels of biomarkers of systemic inflammation (CRP, IL-6, sTNF-αR1 and 2), monocyte activation (CCL2, sCD163, sCD14), coagulation (fibrinogen, D-dimer), and endothelial dysfunction (ICAM-1) with three measures of subclinical carotid atherosclerosis—focal carotid plaque, common carotid IMT (CCA-IMT) and bifurcation-IMT—in the same well-characterized study population of HIV-infected and uninfected men. Our secondary aim was to determine whether these associations differed by HIV serostatus.

## Methods

### Study design, population, and setting

The MACS is an ongoing prospective cohort study of the natural and treated history of HIV infection conducted in Baltimore/Washington DC, Pittsburgh, Los Angeles, and Chicago. Participants are men who have sex with men with or at risk for acquiring HIV infection [20] who enrolled during three periods beginning in 1984. Study procedures have been described previously [20, 21]. Briefly, six-month study visits include standardized interviews, physical examinations, and the collection of blood for concurrent testing and storage in local and national repositories. MACS highlights, protocols, and data collection forms may be found at http://aidscohortstudy.org.

From 2010–2013, the MACS CVD sub-study enrolled participants 40–70 years of age who weighed ≤300 lb and had no prior history of cardiac surgery or percutaneous coronary intervention. Details of the study have been published [22]. A total of 912 participants were evaluated for subclinical carotid atherosclerosis. After excluding participants with missing biomarker, covariate, or carotid data (missing data ranged from 4.4–8.5%), our analytic sample consisted of 728 men (276 HIV-uninfected and 452 HIV-infected). Among them, 672 had evaluable IMT measurements of the bifurcation region. Participants provided written informed consent, and the study was approved by the Institutional Review Boards of all MACS sites: The Chesapeake Institutional Review Board, the Cook County Bureau of Health Services Institutional Review Board, the Johns Hopkins Bloomberg School of Public Institutional Review Board, the Johns Hopkins Medicine Institutional Review Boards, the Los Angeles BioMedical Research Institute at Harbor-UCLA Medical Center John F. Wolf, Human Subjects Committee, the Northwestern University Institutional Review Board, the Ohio State Biomedical Sciences Institutional Review Board, the University of Pittsburgh Institutional Review Board, the University of California Los Angeles Institutional Review Board.

### Main outcome measures

At a separate CVD sub-study visit, participants underwent a high-resolution B mode carotid artery ultrasound at eight different sites in the right carotid artery system using a standardized protocol. The presence of focal carotid plaque at any of these sites was the primary outcome measure, due to its previously reported association with HIV infection in the MACS and the Women’s Interagency HIV Study (WIHS) [1]. The presence of plaque was defined as a localized intima media thickness >1.5 mm [23]. The secondary outcome measures were IMT at the common carotid artery (CCA-IMT) and at the bifurcation of the right branch of the common carotid (bifurcation-IMT), which were quantified using computerized edge tracking multiframe image processing [24]. Study personnel performing the ultrasound received uniform training from the University of Southern California Atherosclerosis Research Unit Core Imaging and Reading Center and were masked to participant HIV serostatus [25].

### Biomarker exposures and other variables

Levels of the ten biomarkers were measured in serum and plasma obtained from blood drawn at the CVD sub-study visit and stored at -70 C. Details of the biomarker assay methods are described in S1 Table [18].

Sociodemographic, behavioral, and traditional cardiovascular risk factors were assessed at the MACS semi-annual study visit. Self-reported sociodemographic variables included age, race/ethnicity, and baseline education. Risk behaviors included self-reported recent alcohol consumption and cigarette smoking, including current smoking status and cumulative pack-years calculated from the baseline cohort visit. Hepatitis C virus infection status (HCV) was determined using antibody and plasma viral RNA levels. Cardiovascular risk factors included measured body mass index (BMI, weight (kg)/height (m)^2^), systolic blood pressure (SBP), fasting glucose levels, total and high-density lipoprotein cholesterol levels (HDL), and self-reported current use of medication for hypertension, diabetes, and high cholesterol.

Positive ELISA confirmed by Western blot determined HIV seropositivity. Plasma HIV RNA (viral load) levels were determined by the Roche ultrasensitive assay, sensitive to 50 copies/mL. Participants were classified as having durable viral suppression over the 5 years prior to the ultrasound scan if viral load measures during this period were <50 copies/mL, with a single blip up to 500 copies/mL permitted. Standardized flow cytometry enumerated CD4^+^ T lymphocyte cell counts/μL (CD4) [26]. The use of highly active antiretroviral therapy (HAART) was self-reported. An AIDS diagnosis was defined clinically by the presence of one or more AIDS-defining illness according to the 1993 CDC criteria (with the exception of CD4< = 200 cells/μL).

### Statistical methods

Differences in distributions of sociodemographic, behavioral, and cardiovascular risk factors by HIV serostatus were assessed using Student *t*-tests or Wilcoxon rank-sum tests for continuous variables and χ^2^ tests for categorical variables. Separate analyses were conducted for each biomarker. Regression analyses were used to evaluate the relationship of each biomarker independently with the presence of focal carotid plaque (logistic regression) and CCA-IMT and bifurcation-IMT (linear regression). Due to their skewed distributions, biomarker levels were divided into quintiles and modeled as categorical variables. Primary results were presented for men with biomarker levels in the highest compared to the lowest quintile. Regression models were first adjusted for sociodemographic and behavioral characteristics: age (years), race/ethnicity (White, non-Hispanic; Black, non-Hispanic; and other), education (did not complete high school, completed high school, any college experience, any graduate school experience), study center, HIV serostatus, and enrollment in the MACS cohort (pre- and post-2001). Subsequent models adjusted for all cardiovascular and behavioral risk factors, including SBP (per 10 mm Hg), BMI (modeled continuously), total and HDL cholesterol levels (per 5 mg/dl), alcohol consumption (none, 1 to 3 drinks/week, 4 to 13 drinks/week and more than 13 drinks/week since last visit), smoking status (never, former, or current) and cumulative pack-year use, and use of medication for hypertension, diabetes and high cholesterol. Analyses conducted among HIV-infected men were further adjusted for contemporaneous HAART use, 5-year viral suppression, current CD4 cell count, the lowest historically-measured CD4 cell count prior to HAART initiation, and history of AIDS diagnoses. Finally, interaction terms assessed whether associations between biomarker levels and each carotid atherosclerosis outcome varied by HIV serostatus.

Statistical significance was determined by a 2-sided p<0.05. Analyses were conducted using Stata, version 14.2 (College Station, Texas).

## Results

### Participant characteristics

Participant characteristics, stratified by HIV serostatus, are depicted in Table 1. Compared to the HIV-uninfected men, HIV-infected participants were younger, more likely to be current smokers, and had lower BMI and HDL cholesterol. Participants were comparable by HIV serostatus with respect to other cardiometabolic factors. Among HIV-infected men, 82.5% were virally suppressed with a median duration of HAART use of 9.2 years. The median current CD4 was 592 cells/mm^3^. About 15% had a history of AIDS, and the median nadir CD4 was 290 cells/mm^3^. Among 184 participants with missing data (excluded from our study sample), the distribution of characteristics remained largely the same (S2 Table).

**Table 1:** Study population characteristics, by HIV serostatus.

Characteristic	HIV-uninfected (n = 276)		HIV-infected (n = 452)	p value
**Demographic factors**				
Age, years	55.5 ± 7.3		52.5 ± 6.6	<0.001
Race/ethnicity				<0.001
White, non-Hispanic	67.8		52.0	
Black, non-Hispanic	23.9		34.7	
Other	8.3		13.3	
Baseline education				<0.001
Did not complete high school	4.7		7.5	
Completed high school	9.4		17.3	
Any college experience	47.5		54.7	
Any graduate school experience	38.4		20.6	
Cohort				<0.001
Pre-2001	65.2		48.7	
Post-2001	34.8		51.3	
**Behavioral risk factors**				
Smoking				<0.01
Never	26.1		23.7	
Former	53.6		44.7	
Current	20.3		31.6	
Cumulative pack year; median (IQR)	0.5 (0, 19.9)		6.9 (0, 22.3)	0.02
Current alcohol use (since last visit)				<0.001
None	13.0		25.0	
1–3 drinks/week	48.6		53.3	
4–13 drinks/week	27.9		16.6	
>13 drinks/week	10.5		5.1	
Current Hepatitis C infection	3.3		10.4	<0.001
**Cardiometabolic risk factors**				
Body mass index, kg/m^2^	27.3 ± 4.5		26.2 ± 4.6	<0.001
Systolic blood pressure, mm Hg	128.8 ± 14.6		126.3 ± 15.1	0.03
Antihypertensive medication	31.2		34.3	0.38
Fasting glucose, mg/dl*	97 (89, 103)		97.5 (90, 107)	0.33
Diabetes medication	7.6		9.5	0.38
Total cholesterol, mg/dl*	195.3 ± 35.8		187.3 ± 41.1	0.01
HDL cholesterol, mg/dl*	51.6 (42.3, 60.3)		46.1 (38.4,54.4)	<0.001
Cholesterol-lowering medication	31.5		34.7	0.37
**HIV-specific factors**				
CD4^+^ T-cell count, cells/mm^3^				
Current			592 (422, 763)	
Nadir			290 (176, 413)	
Current HIV viral suppression (<50 copies/mL)			82.3	
5-year virologic suppression			52.7	
HAART use			88.1	
History of AIDS			15.0	

Abbreviations: HAART, highly active antiretroviral therapy; HDL, high-density lipoprotein; AIDS, Acquired Immune Deficiency Syndrome; CD, cluster of differentiation

Categorical data are described by percent. Continuous data are described by mean and standard deviation (SD) for normally distributed variables or by median and interquartile range (IQR). P values were obtained from Student *t*-tests or Wilcoxon rank-sum tests for continuous variables and χ^2^ tests for categorical variables. Level of significance, p<0.05.

*Multiply by 0.06 (per 1 mg/dl) to obtain glucose and 0.02586 (per 1 mg/dl) to obtain cholesterol levels in mmol/L

The prevalence of focal carotid plaque was 30% in our study population. The median bifurcation-IMT was greater than the median CCA-IMT (0.89 mm vs 0.76 mm) (S1 Fig) and the correlation coefficient was 0.64.

### Inflammatory biomarkers

With the exception of fibrinogen and D-dimer, biomarker levels were significantly higher among HIV-infected men compared with uninfected men, even after adjusting for differences in cardiovascular risk factors (Table 2). The difference persisted when limiting to men with durable virologic suppression over the past 5 years, though differences in levels of CRP and IL-6 became non-significant (S3 Table). The majority of pairwise correlations between biomarkers were weak (<0.3) with correlation coefficients ranging from 0.04 to 0.71 (S4 Table).

**Table 2:** Levels of inflammatory biomarkers, by HIV serostatus.

Biomarker	Cut point for Upper Quintile	HIV-uninfected (n = 276) Median (IQR)	HIV-infected (n = 452) Median (IQR)	Unadjusted p value	Adjusted* p value
sCD163 (ng/ml)	>873	554 (449, 696)	679 (519, 876)	<0.001	<0.001
sCD14 (ng/ml)	>1,810	1,292 (1,130, 1,458)	1,615 (1,393, 1,895)	<0.001	<0.001
ICAM-1 (ng/ml)	>315	228 (192, 270)	258 (215, 314)	<0.001	0.04
CCL2 (pg/ml)	>354	235 (183, 313)	273 (211, 350)	<0.001	<0.001
CRP (ug/ml)	>3.2	1.0 (0.54, 1.98)	1.3 (0.7, 2.8)	0.01	<0.01
IL-6 (pg/ml)	>2.7	1.3 (0.89, 2.17)	1.5 (1.0, 2.4)	0.01	0.01
sTNF-αR1 (pg/ml)	>1,516	1,165 (956, 1,354)	1,167 (950, 1,463)	0.04	0.06
sTNF-αR2 (pg/ml)	>8,188	5,905 (4,961, 6,949)	6,575 (5,436, 8,017)	<0.001	<0.001
Fibrinogen (ml/dl)	>391	336 (294, 380)	326 (282, 377)	0.07	0.11
D-dimer (ug/ml)	>0.4	0.2 (0.13, 0.30)	0.3 (0.1, 0.3)	0.14	0.99

Abbreviations: IQR, interquartile range; sCD163, soluble cluster of differentiation 163; sCD14, soluble cluster of differentiation 14; CCL2, chemokine (C-C motif) ligand 2; ICAM-1, intercellular cell adhesion molecule-1; CRP, C reactive protein; IL-6, interleukin-6; sTNF-αR1, soluble tumor necrosis factor-alpha receptor 1; sTNF-αR2, soluble tumor necrosis factor-alpha receptor 2.

Unadjusted p values were obtained using the Wilcoxon rank sum test.

*Adjusted p values were obtained from Wald tests from multivariable linear regression models that adjusted for age, race/ethnicity, baseline education, study center, enrollment in the MACS cohort (pre- and post-2001), systolic blood pressure, body mass index, total cholesterol, high-density lipoprotein (HDL) cholesterol, alcohol consumption since last visit, smoking status with cumulative pack year use, hepatitis C infection, and use of medication for hypertension, diabetes and high cholesterol.

### Associations of inflammatory biomarkers with focal carotid plaque

**Fig 1:**
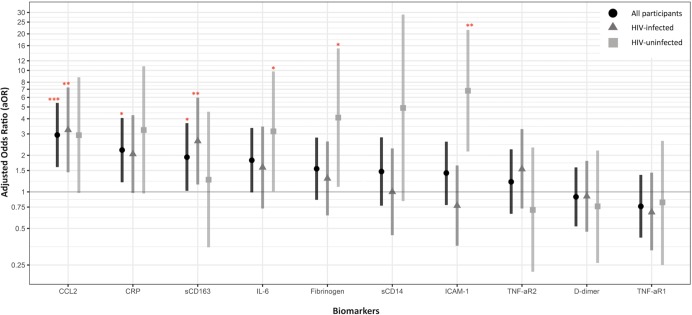
Association of inflammatory biomarkers (fifth quintile, relative to first quintile) with the presence of focal carotid plaque. Models are adjusted for age, race/ethnicity, baseline education, study center, enrollment in the MACS cohort (pre- and post-2001), SBP, BMI, total cholesterol, HDL cholesterol, alcohol consumption since last visit, smoking status with cumulative pack year use, HCV, and use of medication for hypertension, diabetes and high cholesterol. Data points are adjusted odds ratios (aOR), and bars denote 95% confidence intervals (CI). Dark gray horizontal line represents the null value (aOR = 1.00). Level of significance, *p<0.05, **p<0.01, ***p<0.001. Values of the aOR (95% CI), from left to right: All participants (N = 728): CCL2 [2.94 (1.60, 5.40)], CRP [2.21(1.20, 4.06)], sCD163 [1.93 (1.02, 3.68)], IL-6 [1.82 (0.99, 3.36)], Fibrinogen [1.55 (0.86, 2.80)], sCD14 [1.47 (0.77, 2.81)], ICAM-1 [1.43 (0.78, 2.59)], sTNF-αR2 [1.21 (0.66, 2.24)], D-dimer [0.91 (0.52, 1.59)] and sTNF-αR1 [0.76 (0.42, 1.38)]. HIV-infected (N = 452): CCL2 [3.24 (1.45, 7.24)], CRP [2.05 (0.98, 4.28)], sCD163 [2.62 (1.15, 5.97)], IL-6 [1.59 (0.73, 3.45)], Fibrinogen [1.29 (0.64, 2.60)], sCD14 [1.00 (0.44, 2.28)], ICAM-1 [0.77 (0.36, 1.65)], sTNF-αR2 [1.54 (0.72, 3.29)], D-dimer [0.92 (0.47, 1.80)] and sTNF-αR1 [0.68 (0.33, 1.44)]. HIV-uninfected (N = 276): CCL2 [2.93 (0.98, 8.78)], CRP [3.23 (0.97, 10.82)], sCD163 [1.25 (0.35, 4.570], IL-6 [3.15 (1.01, 9.81)], Fibrinogen [4.09 (1.10, 15.18)], sCD14 [4.91 (0.84, 28.82)], ICAM-1 [6.8 (2.15, 21.54)], sTNF-αR2 [0.71(0.22, 2.32)], D-dimer [0.76 (0.26, 2.19)] and sTNF-αR1 [0.82 (0.25, 2.63)].

In the overall study population, higher levels of 8 of the 10 biomarkers were significantly associated with a greater odds of focal carotid plaque in unadjusted analyses; four biomarkers—sCD163, CCL2, CRP, and IL-6—remained significantly or nearly significantly associated after adjustment for demographic, behavioral, and traditional CVD factors (Fig 1). Generally, the odds of plaque were greater with each increasing quintile of biomarker levels, consistent with a dose-response relationship (S5 Table). Higher levels of CCL2 (quintile 5, relative to quintile 1) were associated with nearly three times the odds of focal carotid plaque in fully-adjusted models (aOR = 2.94, p<0.001). Higher levels of sCD163 (aOR = 1.93, p = 0.04), CRP (aOR = 2.21, p = 0.01), and IL-6 (aOR = 1.82, p = 0.05) were also positively associated with carotid plaque. With the exception of IL-6, when the biomarkers were all included in a single, fully-adjusted model these findings generally persisted: CCL2 (aOR = 3.16, p = 0.001), CRP (aOR = 2.27, p = 0.04), and sCD163 (aOR = 2.0, p = 0.05) (S6 Table). The statistical significance of the associations with focal plaque observed for sCD14, ICAM-1, sTNF-αR2, and fibrinogen in unadjusted analyses was attenuated in fully-adjusted models, while levels of TNF-αR1 and D-dimer were not associated with carotid plaque.

In stratified analyses (Fig 1, S7 Table), among HIV-infected men, higher levels of CCL2 and sCD163 were associated with greater odds of prevalent focal carotid plaque after adjusting for demographic and cardiovascular factors (aOR = 3.24, p<0.01 and aOR = 2.62, p = 0.02, respectively). The association of elevated CRP levels with focal plaque did not reach statistical significance (aOR = 2.05, p = 0.06). These associations persisted, and, in the case of sCD163 and CRP, increased in magnitude (aOR = 3.39, p = 0.01 and aOR = 2.26, p = 0.04, respectively), after additional adjustment for CD4, CD4 nadir, use of HAART, history of AIDS diagnosis, and 5-year HIV viral suppression (S7 Table). These inferences were unchanged in sensitivity analyses restricted to men with virologic suppression (S8 Table).

Among the HIV-uninfected men, higher levels CCL2 and CRP were also associated with focal carotid plaque at a similar magnitude, though not statistically significantly. However, there was no association between focal carotid plaque with sCD163. The association between IL-6 levels and focal carotid plaque was larger in magnitude among HIV-uninfected men (aOR = 3.15, p = 0.05) than the HIV-infected men (aOR = 1.59, p = 0.24), though the effect measure modification was not significant on the multiplicative scale. Higher levels of ICAM-1 and fibrinogen were associated with significantly higher odds of focal carotid plaque (aOR = 6.80, p = 0.001 and aOR = 4.09, p = 0.04, respectively). ICAM-1 was the only biomarker whose association with carotid plaque differed significantly by HIV serostatus, with an association apparent only among the HIV-uninfected men (interaction term p<0.001).

### Associations of inflammatory biomarkers with common carotid IMT

In the overall study population, higher levels of six biomarkers (sTNF-αR1, sTNF-αR2, IL-6, CCL2, CRP, and fibrinogen) were significantly associated with greater CCA-IMT in unadjusted analyses, with mean differences ranging from β = 0.03 mm to 0.07 mm for biomarker levels in the highest relative to the lowest quintile (Table 3A, S9 Table). Only fibrinogen remained positively associated with CCA-IMT after adjustment for sociodemographic and CVD risk factors (β = 0.04 mm, p = 0.01). Conversely, the fifth (highest) quintile of D-dimer was inversely associated with CCA-IMT (β = 0.04 mm, p = 0.01) after adjusting for sociodemographic and CVD risk factors. There were no associations between levels of sCD14, sCD163, and ICAM-1 and CCA-IMT.

**Table 3:** Associations between inflammatory biomarkers and intima media thickness of the right common carotid artery (CCA-IMT) (N = 728).

Biomarkers	Model A*			Model B^†^			Model C^‡^		
	β (95% CI)		p value	β (95% CI)		p value	β (95% CI)		p value
sCD163	0.03	[-0.00, 0.06]	0.06	0.01	[-0.02, 0.04]	0.48	0.01	[-0.03, 0.04]	0.74
sCD14	0.03	[-0.01, 0.06]	0.11	0.01	[-0.02, 0.05]	0.42	0.01	[-0.02, 0.05]	0.54
ICAM-1	0.02	[-0.01, 0.06]	0.13	0.02	[-0.02, 0.05]	0.33	0.00	[-0.03, 0.04]	0.81
CCL2	**003**	**[0.00, 0.07]**	**0.04**	0.02	[-0.01, 0.05]	0.26	0.01	[-0.02, 0.04]	0.58
CRP	**0.05**	**[0.02, 0.08]**	**<0.01**	**0.03**	**[0.00, 0.06]**	**0.03**	0.02	[-0.02, 0.05]	0.32
IL-6	**0.05**	**[0.02, 0.08]**	**<0.01**	0.02	[-0.01, 0.06]	0.13	0.00	[-0.03, 0.04]	0.80
sTNF-αR1	**0.05**	**[0.01, 0.08]**	**<0.01**	0.03	[-0.00, 0.06]	0.09	0.02	[-0.01, 0.05]	0.19
sTNF-αR2	**0.05**	**[0.02, 0.09]**	**<0.001**	0.03	[-0.00, 0.06]	0.06	0.03	[-0.00, 0.06]	0.08
Fibrinogen	**0.07**	**[0.04, 0.11]**	**<0.001**	**0.06**	**[0.02, 0.09]**	**<0.001**	**0.04**	**[0.01, 0.07]**	**0.01**
D-dimer	0.00	[-0.03, 0.03]	0.92	**-0.03**	**[-0.06, -0.00]**	**0.02**	**-0.04**	**[-0.06, -0.01]**	**0.01**

Abbreviations: sCD163, soluble cluster of differentiation 163; sCD14, soluble cluster of differentiation 14; CCL2, chemokine (C-C motif) ligand 2;

ICAM-1, intercellular cell adhesion molecule-1; CRP, C reactive protein; IL-6, interleukin-6; sTNF-αR1, soluble tumor necrosis factor-

alpha receptor 1; sTNF-αR2, soluble tumor necrosis factor-alpha receptor 2. Results are presented as β coefficients and 95% CI. Bolded results are significant (p<0.05).

*Model A: Unadjusted

†Model B: Adjusted for HIV status, age, race/ethnicity, baseline education, study center, enrollment period in the MACS cohort (pre- and post-2001)

‡Model C: Adjusted for variables in model B and systolic blood pressure, body mass index, total cholesterol, high-density lipoprotein (HDL) cholesterol, alcohol consumption since last visit, smoking status with cumulative pack year use, hepatitis C infection, and use of medication for hypertension, diabetes and high cholesterol

Among the HIV-infected men, fibrinogen levels were significantly associated with CCA-IMT in the minimally-adjusted models but the association disappeared after further adjustment for CVD risk factors (S10 Table). Among the HIV-uninfected men, both fibrinogen and sTNF-αR2 levels were significantly associated with CCA-IMT in fully-adjusted models, while ICAM-1 and CRP were significantly associated in minimally-adjusted models (p = 0.03 and p = 0.01) but not after adjusting for traditional CVD risk factors.

### Associations of inflammatory biomarkers with bifurcation region IMT

In the overall cohort, levels of six biomarkers were significantly associated with bifurcation-IMT in unadjusted analyses but only three remained significant in fully-adjusted models: ICAM-1 (β = 0.05 mm, p = 0.02), CCL2 (β = 0.04 mm, p = 0.05), and sTNF-αR1 (β = 0.04 mm, p = 0.05) (Table 4, S11 Table). The magnitudes of these associations were similar after stratification by HIV serostatus, in both strata, but were not statistically significant (S12 Table).

**Table 4:** Associations between inflammatory biomarkers and intima media thickness at the bifurcation of the right common carotid artery (bifurcation-IMT) (N = 672).

Biomarkers	Model A*			Model B^†^			Model C^‡^		
	β (95% CI)		p value	β (95% CI)		p value	β (95% CI)		p value
sCD163	0.03	[-0.01, 0.07]	0.21	0.02	[-0.02, 0.06]	0.36	0.02	[-0.02, 0.07]	0.27
sCD14	0.01	[-0.03, 0.05]	0.66	0.01	[-0.04, 0.05]	0.77	0.01	[-0.03, 0.05]	0.64
ICAM-1	**0.05**	**[0.01, 0.09]**	**0.01**	**0.06**	**[0.02, 0.10]**	**<0.01**	**0.05**	**[0.01, 0.09]**	**0.02**
CCL2	**0.06**	**[0.02, 0.10]**	**<0.01**	**0.05**	**[0.01, 0.09]**	**0.02**	**0.04**	**[0.00, 0.08]**	**0.05**
CRP	**0.04**	**[0.00, 0.08]**	**0.03**	0.03	[-0.01, 0.07]	0.10	0.02	[-0.02, 0.05]	0.44
IL-6	**0.06**	**[0.02, 0.09]**	**0.01**	0.03	[-0.01, 0.07]	0.14	0.02	[-0.02, 0.06]	0.43
sTNF-αR1	**0.05**	**[0.01, 0.09]**	**0.01**	0.04	[-0.00, 0.08]	0.06	**0.04**	**[0.00, 0.08]**	**0.05**
sTNF-αR2	0.03	[-0.01, 0.07]	0.13	0.01	[-0.03, 0.05]	0.60	0.02	[-0.02, 0.06]	0.42
Fibrinogen	**0.07**	**[0.03, 0.11]**	**<0.001**	**0.05**	**[0.01, 0.09]**	**0.02**	0.03	[-0.01, 0.07]	0.11
D-dimer	0.02	[-0.02, 0.06]	0.29	-0.02	[-0.06, 0.01]	0.22	-0.02	[-0.06, 0.02]	0.25

Abbreviations: sCD163, soluble cluster of differentiation 163; sCD14, soluble cluster of differentiation 14; CCL2, chemokine (C-C motif) ligand 2;

ICAM-1, intercellular cell adhesion molecule-1; CRP, C reactive protein; IL-6, interleukin-6; sTNF-αR1, soluble tumor necrosis factor-

alpha receptor 1; sTNF-αR2, soluble tumor necrosis factor-alpha receptor 2. Results are presented as β coefficients and 95% CI. Bolded results are significant (p<0.05).

*Model A: Unadjusted

†Model B: Adjusted for HIV status, age, race/ethnicity, baseline education, study center, cohort

‡Model C: Adjusted for variables in model B and systolic blood pressure, body mass index, total cholesterol, high-density lipoprotein (HDL) cholesterol, alcohol consumption since last visit, smoking status with cumulative pack year use, hepatitis C infection, and use of medication for hypertension, diabetes and high cholesterol

Among HIV-uninfected men, higher levels of fibrinogen were positively associated with bifurcation-IMT (p<0.05), but this was not observed in HIV-infected men (interaction term p = 0.08). No significant associations between sCD163, sCD14, D-dimer, or sTNF-αR2 with bifurcation-IMT were observed.

## Discussion

In this large, well-characterized cohort of HIV-infected and otherwise similar uninfected men who were assessed for subclinical carotid atherosclerosis, we found that HIV-infected men, even those with durable virologic suppression, had significantly higher levels of nearly all evaluated biomarkers. Furthermore, we found that elevated levels of two monocyte activation biomarkers, CCL2 and sCD163, and two systemic inflammation biomarkers, CRP and IL-6, were significantly (borderline for IL-6) associated with the presence of focal carotid plaque, independent of sociodemographic and traditional CVD risk factors. Fibrinogen levels were positively associated with CCA-IMT while ICAM-1, CCL2, and sTNF-αR1 were positively associated with bifurcation-IMT, our secondary measures of subclinical carotid atherosclerosis. In stratified analyses, associations with focal carotid plaque were generally consistent by HIV serostatus, with the exception of a large measure of association for monocyte activation marker sCD163 among the HIV-infected but not the uninfected, and stronger associations with ICAM-1, IL-6, and fibrinogen among the uninfected. Differences by HIV serostatus were more evident for associations with the IMT measures, with significant associations observed only among the HIV-uninfected men.

Our findings provide additional support for a primary role of monocyte activation in vascular plaque pathogenesis, particularly among HIV-infected individuals. The biomarker with the most robust association with subclinical carotid atherosclerosis in the present study, based on magnitude, consistency of associations regardless of HIV serostatus, and coherence across carotid measures, was CCL2. A chemokine involved primarily in regulating the migration and infiltration of monocytes, CCL2 has been implicated as an essential mediator in the pathogenesis of atherosclerotic plaque formation, [27] and has been associated with carotid, femoral, [28–30] and thoracic aortic atherosclerosis [31, 32]. Additionally, consonant with prior studies, our results support the role of sCD163 in atherosclerosis among HIV-infected persons [33–39]. sCD163 is shed from the cell surface upon monocyte/macrophage activation, a central component of the atherosclerotic process [40]. In the present analysis, higher CCL2 and sCD163 levels were each associated with over 3 times the odds of focal carotid plaque in HIV-infected men, independent of traditional demographic, CVD risk factors, and HIV-related characteristics. A previous study in the MACS found that elevated CCL2 and sCD163 levels were associated with subclinical coronary atherosclerosis [18]. That CCL2 and sCD163 were associated with atherosclerotic plaque at both coronary and carotid sites in this cohort of HIV-infected and uninfected men provides valuable evidence of its role in subclinical atherosclerosis [41].

Despite the signal between the monocyte activation markers and carotid atherosclerosis, the fact that CCL2 and sCD163 were associated with carotid plaque but not with CCA-IMT in our study is, to our knowledge, a novel finding. This pattern of association is congruent with previous associations observed between HIV infection and carotid plaque, but not CCA-IMT, providing further coherence to the hypothesis that monocyte activation is one mechanism underlying the increased risk of subclinical atherosclerosis among HIV-infected individuals Similarly, the differential association of CCL2 with bifurcation IMT, but not CCA-IMT, has not been previously reported to our knowledge. The progression of atherosclerosis among HIV-infected individuals has been found to be more pronounced in the bifurcation region compared to the common carotid artery [10].

While substantial evidence has demonstrated that chronic generalized inflammation plays a pivotal role in the pathogenesis of atherosclerosis, [14] the associations between levels of systemic biomarkers of inflammation and subclinical atherosclerosis have not been fully characterized, especially among HIV-infected persons. In the current study, HIV-infected men had significantly higher levels of CRP, IL-6, and ICAM-1 than the uninfected men, although among those with durable virologic suppression only ICAM-1 remained significantly higher. Higher levels of CRP and IL-6 were associated with increased odds of carotid focal plaque even after adjusting for traditional CVD risk factors, though some of the HIV-stratified results were of borderline significance. Previously, we observed that higher levels of IL-6, but not CRP, were associated with increased prevalence of coronary stenosis ≥50% and greater coronary artery calcification score among HIV-infected men in the MACS [19]. These findings provide further evidence of the role of generalized inflammation in the development of both subclinical carotid and coronary atherosclerosis. Conversely, no associations between CRP and IL-6 levels with IMT were observed; this finding diverges from previous reports in which elevated CRP was associated with progressive wall thickening of the carotid bifurcation region among HIV-infected patients [42] and higher levels of IL-6 were associated with overall and bifurcation-IMT [43]. There appears to be a more consistent association between CRP, IL-6, and D-dimer and CVD events compared to with subclinical measures such as IMT in HIV-infected populations [14].

In contrast to the other markers of inflammation, elevated levels of ICAM-1 and fibrinogen were associated with focal plaque only among HIV-uninfected men. Fibrinogen, which mediates leukocyte adhesion through an ICAM-1-dependent pathway [44], was also associated with greater CCA-IMT and bifurcation-IMT among HIV-uninfected men, independent of CVD risk factors. This consistency across all three plaque measures corroborates previous studies associating fibrinogen with subclinical atherosclerosis measures, including increased CCA-IMT [45–47] and coronary artery calcification [47, 48] in the general population. Elevated levels of ICAM-1 were also strongly associated with higher odds of focal plaque but not CCA-IMT among the HIV-uninfected men, a pattern supported by some previous studies [49, 50] but not by others [51–54]. Inconsistent findings in the literature may reflect methodological differences, different study populations, or divergent pathophysiologies. Furthermore, it is unclear why the relationships observed between ICAM-1 and fibrinogen with carotid markers of disease among uninfected men were not seen among the HIV-infected men. Complex immune dysregulation associated with HIV infection, such as its influence on hepatocyte protein synthesis and inflammation [55, 56], may be involved in this discordance.

Taken together, our findings provide further evidence for the role of generalized inflammation, as well as specific monocyte activation pathways, in subclinical atherosclerosis, with the latter having greater significance among HIV-infected individuals. The implications for prevention include the potential for a greater role of statins, which, despite their effectiveness in reducing cholesterol and immune activation in the general population, remain under study in the context of HIV infection and concurrent ART use in the ongoing REPRIEVE trial. In addition, therapy targeting specific inflammatory pathways are being evaluated. Notably, the CANTOS trial demonstrated that Canakinumab, an inhibitor of the interleukin-1β inflammatory pathway, lowered the rate of recurrent cardiovascular events, independent of cholesterol-lowering medication [57]. Though in its earliest stages, preliminary experimental and preclinical studies suggest that targeted pharmacological agents may effectively regulate monocyte activation pathways and have the potential to inhibit the progression of atherosclerosis, which might be particularly relevant for HIV-infected populations [58].

The present study has limitations. First, the cross-sectional design precludes the ability to make temporal inferences about relationships between biomarker levels and the carotid outcome measures, though a prior study of HIV-infected men in the MACS found that levels of many of these biomarkers do not change appreciably after the first year of HAART-induced viral suppression [59, 60]. Given that some of the CVD risk factors are also implicated in inflammatory pathways (e.g. BMI, cholesterol), regression models adjusting for these factors may have over-adjusted, potentially attenuating the observed associations with inflammatory biomarker levels and rendering the presented estimates conservative. Moreover, the male study population may preclude the generalizability of these findings to women. Finally, our study evaluated the associations between multiple biomarkers and markers of subclinical carotid atherosclerosis, and it is possible some findings were significant solely due to chance. Results of borderline statistical significance should be interpreted cautiously with consideration regarding the magnitude of the reported measure of association and be further evaluated in other populations.

This study has several strengths. The MACS is a large, well-characterized cohort study of both HIV-infected and uninfected men similar in sociodemographic and other risk factor profiles, many of whom have been followed for more than 25 years with in-depth and standardized data collection, specimen collection, processing, and storage. In addition, this study evaluated a diverse selection of biomarkers representing different inflammatory, immune activation, and vascular domains, allowing us to investigate whether multiple pathways are implicated in subclinical atherosclerosis in this population. Finally, we conducted a detailed assessment of subclinical carotid atherosclerosis utilizing three measures obtained via high-resolution ultrasound, including intima media thickness at the branching of the right carotid artery, a location where carotid atherosclerosis has been found to progress more rapidly among HIV-infected persons [10]. The consistent associations across, and distinctions among, the three carotid sites indicate the value of including additional measurements beyond CCA-IMT to extend our understanding of the role of inflammation in carotid atherosclerosis.

In conclusion, levels of biomarkers of monocyte activation, systemic inflammation, and endothelial dysfunction were significantly elevated among HIV-infected men, and seven of these inflammatory markers were associated with subclinical carotid atherosclerosis independent of traditional cardiovascular risk factors in a large sample of HIV-infected and HIV-uninfected men. These findings, taken together with previously reported associations of levels of these markers with coronary atherosclerosis in the same cohort, provide further evidence that monocyte activation, in addition to generalized inflammation pathways, contribute to subclinical atherosclerosis, especially in the context of HIV-related immune dysregulation. The observed association between monocyte activation markers and carotid focal plaque, but not CCA-IMT, is unique and supplements evidence that these biomarkers may explain some of the increased risk of subclinical atherosclerosis in HIV infection. Ongoing longitudinal studies will clarify these relationships and investigate the utility of inflammatory biomarker level measurement for disease risk stratification and as targets for prophylactic or therapeutic interventions.

## Supporting information

S1 Fig Intima media thickness (in mm) at the right common carotid artery (CCA-IMT) and at the bifurcation of the right common carotid artery (bifurcation-IMT). (TIFF)

S1 Table Biomarker assay description and coefficient of variation. (PDF)

S2 Table Comparison of study participants included in the analytic sample to those excluded due to missing biomarker or covariate values. (PDF)

S3 Table Levels of inflammatory biomarkers, by HIV serostatus and level of viral suppression. (PDF)

S4 Table Correlation between inflammatory biomarkers, stratified by HIV serostatus. (PDF)

S5 Table Associations between inflammatory biomarkers and the presence of focal carotid plaque (N = 728). (PDF)

S6 Table Adjusted associations between inflammatory biomarkers (mutually adjusted for each other) and focal carotid plaque (N = 728). (PDF)

S7 Table Associations between inflammatory biomarkers and focal carotid plaque, by HIV serostatus (n = 728). (PDF)

S8 Table Associations between biomarkers and markers of subclinical carotid atherosclerosis among HIV-infected men with suppressed viral load. (PDF)

S9 Table Associations between inflammatory biomarkers and intima media thickness at the right common carotid artery (N = 728). (PDF)

S10 Table Associations between the fifth quintile of the inflammatory biomarkers and intima media thickness at the right common carotid artery, by HIV serostatus (n = 728). (PDF)

S11 Table Associations between inflammatory biomarkers and IMT at the bifurcation of the right common carotid artery (N = 672). (PDF)

S12 Table Associations between the fifth quintile of the inflammatory biomarkers and intima media thickness at the bifurcation of the right common carotid artery, by HIV serostatus (n = 672). (PDF)
