# Supplementary material for: Inflammatory biomarkers and subclinical carotid atherosclerosis in HIV-infected and HIV-uninfected men in the Multicenter AIDS Cohort Study
Source: PLoS One. 2019 Apr 4;14(4):e0214735. doi: 10.1371/journal.pone.0214735 (PMC6448851; doi:10.1371/journal.pone.0214735)
Supplement: S1 Table — (PDF) [file pone.0214735.s002.pdf]

S1 Table. Biomarker assay description and coefficient of variation

Biomarker	Sample source	Assay	CV
sCD163	Serum	ELISA method (R&D Systems).	1.7-5.8%
sCD14	Plasma	ELISA method (R&D Systems)	3.96-5.2%
ICAM-1	Serum	Chemiluminescent ELISA method (R&D Systems, Minneapolis, MN)	3.3-7%
CCL2	Plasma	Luminex-based singleplex cytokine panel (Millipore, catalog no. MPXHCYTO60K-01)	3.2-4.1%
CRP	Plasma	BNII Nephelometer Siemens (Siemens Healthcare Diagnostics, Deerfield, IL)	4.5-5.2%
IL-6	Serum	Chemiluminescent ELISA method (R&D Systems, Minneapolis, MN)	7-12%
sTNF-αR1	Plasma (EDTA)	Milliplex soluble cytokine receptor panel (Millipore, Billerica, MA)	4.6-10.8%
sTNF-αR2	Plasma (EDTA)	Milliplex soluble cytokine receptor panel (Millipore, Billerica, MA)	4.2-7.9%
Fibrinogen	Plasma (EDTA)	BNII Nephelometer Siemens (Siemens Healthcare Diagnostics, Deerfield, IL)	1.0-6.7%
D-dimer	Plasma (Citrated)	Stago (Parsippany, NJ) STA-R analyzer	4-17%

Abbreviations: sCD163, soluble cluster of differentiation 163; sCD14, soluble cluster of differentiation 14; ICAM-1, intercellular cell adhesion molecule-1; CCL2, chemokine (C-C motif) ligand 2; CRP, C reactive protein; IL-6, interleukin-6; sTNF-αR1, soluble tumor necrosis factor-alpha receptor 1; sTNF-αR2, soluble tumor necrosis factor-alpha receptor 2.
