# Supplementary material for: Inflammatory biomarkers and subclinical carotid atherosclerosis in HIV-infected and HIV-uninfected men in the Multicenter AIDS Cohort Study
Source: PLoS One. 2019 Apr 4;14(4):e0214735. doi: 10.1371/journal.pone.0214735 (PMC6448851; doi:10.1371/journal.pone.0214735)
Supplement: S2 Table — (PDF) [file pone.0214735.s003.pdf]

S2 Table. Comparison of study participants included in the analytic sample to those excluded due to missing biomarker or covariate values.

Characteristic	Participants with complete data		Participants with missing data	
	HIV-uninfected (N=276)	HIV-infected (N=452)	HIV-uninfected (N=76)	HIV-infected (N=108)
**Demographic risk factors**				
Age, years	55.5 ± 7.3	52.5 ± 6.6	54.5 ± 7.6	53.1 ± 6.2
Race/ethnicity				
White, non-Hispanic	67.8	52.0	64.5	57.4
Black, non-Hispanic	23.9	34.7	29.0	26.9
Other	8.3	13.3	6.6	15.7
Baseline education				
Did not complete high school	4.7	7.5	4.0	8.3
Completed high school	9.4	17.3	18.4	13.9
Some college or completed college	47.5	54.7	47.4	48.2
Attended/completed graduate school	38.4	20.6	30.3	29.6
Cohort				
Pre 2001	65.2	48.7	54.0	50.0
2001 onwards	34.8	51.3	46.1	50.0
**Behavioral risk factors**				
Smoking				
Never	26.1	23.7	24.7	32
Former	53.6	44.7	48	38.8
Current	20.3	31.6	27.4	29.1
Current alcohol use (since last visit)				
Abstain	13	25	21.9	25.7

Characteristic	Participants with complete data		Participants with missing data	
	HIV-uninfected (N=276)	HIV-infected (N=452)	HIV-uninfected (N=76)	HIV-infected (N=108)
Light (1-3 drinks/ week)	48.6	53.3	48	46.5
Moderate (4-13 drinks/week)	27.9	16.6	21.9	20.8
Heavy (>13 drinks/week)	10.5	5.1	8.2	6.9
Current Hepatitis C infection	3.3	10.8	9.3	11.9
**Cardio metabolic risk factors**				
Body Mass Index (kg/m^2)	27.3 $\pm$ 4.5	26.2 $\pm$ 4.6	27.8 $\pm$ 5.6	26.2 $\pm$ 4.4
Systolic blood pressure, mm Hg	128.8 $\pm$ 14.6	126.3 $\pm$ 15.1	127.1 $\pm$ 15.9	128 $\pm$ 15.1
Hypertensive medication	31.2	34.3	30.6	39.2
Fasting glucose, mg/dl	97 (89, 103)	97.5 (90, 107)	97 (90, 107)	101 (95, 110)
Diabetes medication	7.6	9.5	5.8	7.7
Total cholesterol, mg/dl	195.3 $\pm$ 35.8	187.3 $\pm$ 41.1	189.5 $\pm$ 41.2	197.2 $\pm$ 45.0
HDL cholesterol, mg/dl	51.6 (42.3, 60.3)	46.1 (38.4, 54.4)	49.9 (40.4, 58.6)	43.2 (36.9, 53.5)
Cholesterol medication	31.5	34.7	23.5	43.8
**HIV risk factors**				
CD4+ T cell count, cells/mm^3				
Current		592 (422, 763)		636 (466, 754)
Nadir		290 (176, 413)		285 (160, 408)
Current undetectable viral load (< 50 copies/mL)		82.3		80.2
5-year virologic suppression		47.4		45.2
HAART use		88.1		87.0
History of AIDS		15.0		13.0

Abbreviations: HAART, highly active antiretroviral therapy; HDL, high-density lipoprotein; AIDS, Acquired Immune Deficiency Syndrome; BMI, body mass index; CD, cluster of differentiation. Categorical data are described by percent. Continuous data are described by mean and standard deviation (SD) for normally distributed variables, else by median and interquartile range (IQR). P values were obtained using t-test or the chi-square test. Level of significance, $p < 0.05$.
