# Supplementary material for: Inflammatory biomarkers and subclinical carotid atherosclerosis in HIV-infected and HIV-uninfected men in the Multicenter AIDS Cohort Study
Source: PLoS One. 2019 Apr 4;14(4):e0214735. doi: 10.1371/journal.pone.0214735 (PMC6448851; doi:10.1371/journal.pone.0214735)
Supplement: S3 Table — (PDF) [file pone.0214735.s004.pdf]

S3 Table. Levels of inflammatory biomarkers, by HIV serostatus and level of viral suppression

Biomarker	HIV negative (n= 276) Median (IQR)	HIV positive suppressed (n= 238) Median (IQR)	Unadjusted P-value
sCD163 (ng/ml)	553.7 (449, 695.7)	656.1 (505.9, 837.8)	**<0.001**
sCD14 (ng/ml)	1291.9 (1130.1, 1457.5)	1624.7 (1395.7, 1895)	**<0.001**
ICAM-1 (ng/ml)	228.3 (192.1, 269.9)	251.5 (213.3, 301.5)	**<0.001**
CCL2 (pg/ml)	235 (182.5, 312.7)	272.1 (215.0, 350.5)	**<0.001**
CRP (ug/ml)	1.0 (0.5, 2)	1.2 (0.7, 2.8)	**0.07**
IL-6 (pg/ml)	1.3 (0.9, 2.2)	1.5 (1.0, 2.3)	**0.46**
sTNF-αR1 (pg/ml)	1165.1 (956.4, 1354.4)	1182.4 (986.2, 1472.5)	**0.04**
sTNF-αR2 (pg/ml)	5904.9 (4961, 6949.1)	6344.5 (5354.9, 7673.4)	**0.02**
Fibrinogen (ml/dl)	0.2 (0.1, 0.3)	0.2 (0.1, 0.3)	**0.06**

Abbreviations: IQR, interquartile range; sCD163, cluster of differentiation 163; sCD14, cluster of differentiation 14; ICAM-1, intercellular cell adhesion molecule-1; CCL2, chemokine (C-C motif) ligand 2; CRP, C reactive protein; IL-6, interleukin-6; sTNF-αR1, tumor necrosis factor-alpha receptor 1; sTNF-αR2, tumor necrosis factor-alpha receptor 2. Unadjusted p-values were obtained using the Wilcoxon rank sum test.
