# Supplementary material for: Inflammatory biomarkers and subclinical carotid atherosclerosis in HIV-infected and HIV-uninfected men in the Multicenter AIDS Cohort Study
Source: PLoS One. 2019 Apr 4;14(4):e0214735. doi: 10.1371/journal.pone.0214735 (PMC6448851; doi:10.1371/journal.pone.0214735)
Supplement: S4 Table — (PDF) [file pone.0214735.s005.pdf]

S4 Table. Correlation among inflammatory biomarkers, stratified by HIV serostatus

HIV-infected men (N=452)										
	**sCD163**	**sCD14**	**CCL2**	**IL-6**	**ICAM-1**	**Fibrinogen**	**D-dimer**	**CRP**	**sTNF-αR1**	**sTNF-αR2**
**sCD163**	1.00									
**sCD14**	0.12	1.00								
**CCL2**	0.15	0.19	1.00							
**IL-6**	0.10	0.14	0.05	1.00						
**ICAM-1**	0.46	0.21	0.16	0.15	1.00					
**Fibrinogen**	0.06	0.12	0.10	0.12	0.06	1.00				
**D-dimer**	0.13	0.20	0.05	0.21	0.12	0.11	1.00			
**CRP**	0.04	0.27	0.16	0.28	0.15	0.21	0.45	1.00		
**sTNF-αR1**	0.14	0.21	0.25	0.10	0.12	0.06	0.16	0.09	1.00	
**sTNF-αR2**	0.42	0.38	0.33	0.24	0.43	0.13	0.33	0.35	0.71	1.00
**HIV-uninfected men (N=276)**										
	**sCD163**	**sCD14**	**CCL2**	**IL-6**	**ICAM-1**	**Fibrinogen**	**D-dimer**	**CRP**	**sTNF-αR1**	**sTNF-αR2**
**sCD163**	1.00									
**sCD14**	0.08	1.00								
**CCL2**	0.06	0.07	1.00							
**IL-6**	0.15	0.23	0.30	1.00						
**ICAM-1**	0.34	0.06	0.05	0.11	1.00					
**Fibrinogen**	0.18	0.21	0.15	0.26	0.16	1.00				
**D-dimer**	0.06	0.16	0.23	0.44	0.06	0.26	1.00			
**CRP**	0.18	0.28	0.22	0.50	0.19	0.52	0.47	1.00		
**sTNF-αR1**	0.21	0.11	0.29	0.28	0.17	0.17	0.27	0.20	1.00	
**sTNF-αR2**	0.35	0.21	0.22	0.21	0.34	0.17	0.09	0.25	0.61	1.00

Pearson correlation coefficient included in tables with 1.0 indicating a perfect positive correlation. Abbreviations: sCD163, cluster of differentiation 163; sCD14, cluster of differentiation 14; CCL2, chemokine (C-C motif) ligand 2; IL-6, interleukin-6; ICAM-1, intercellular cell adhesion molecule-1; CRP, C reactive protein; sTNF-αR1, tumor necrosis factor-alpha receptor 1; sTNF-αR2, tumor necrosis factor-alpha receptor 2.
