# Supplementary material for: Inflammatory biomarkers and subclinical carotid atherosclerosis in HIV-infected and HIV-uninfected men in the Multicenter AIDS Cohort Study
Source: PLoS One. 2019 Apr 4;14(4):e0214735. doi: 10.1371/journal.pone.0214735 (PMC6448851; doi:10.1371/journal.pone.0214735)
Supplement: S5 Table — (PDF) [file pone.0214735.s006.pdf]

S5 Table. Associations between inflammatory biomarkers and the presence of focal carotid plaque (N=728)

Biomarker	Model A* OR (95% CI)		Model B† aOR (95% CI)		Model C‡ aOR (95% CI)	
**sCD163**						
Quintile 1	Ref ‖	Ref	Ref ‖	Ref	Ref ‖	Ref
Quintile 2	1.15	[0.67, 1.95]	1.08	[0.61, 1.90]	1.18	[0.65, 2.15]
Quintile 3	1.46	[0.87, 2.47]	1.31	[0.75, 2.31]	1.49	[0.81, 2.71]
Quintile 4	1.51	[0.90, 2.55]	1.39	[0.78, 2.46]	1.45	[0.79, 2.69]
Quintile 5	2.39***	[1.43, 3.99]	1.97*	[1.12, 3.47]	1.93*	[1.02, 3.68]
**sCD14**						
Quintile 1	Ref ‖	Ref	Ref ‖	Ref	Ref	Ref
Quintile 2	0.73	[0.43, 1.25]	0.81	[0.46, 1.43]	0.84	[0.46, 1.54]
Quintile 3	1.18	[0.71, 1.96]	1.12	[0.64, 1.94]	1.03	[0.58, 1.85]
Quintile 4	1.18	[0.71, 1.96]	1.19	[0.67, 2.13]	1.12	[0.61, 2.08]
Quintile 5	2.00**	[1.22, 3.27]	2.01*	[1.11, 3.62]	1.47	[0.77, 2.81]
**ICAM-1**						
Quintile 1	Ref ‖	Ref	Ref	Ref	Ref	Ref
Quintile 2	1.26	[0.76, 2.08]	1.02	[0.59, 1.78]	0.96	[0.53, 1.71]
Quintile 3	0.98	[0.58, 1.66]	0.78	[0.44, 1.40]	0.78	[0.42, 1.45]
Quintile 4	1.22	[0.73, 2.04]	0.78	[0.44, 1.37]	0.66	[0.36, 1.20]
Quintile 5	2.21**	[1.35, 3.62]	1.90*	[1.10, 3.26]	1.43	[0.78, 2.59]
**CCL2**						
Quintile 1	Ref ‖	Ref	Ref ‖	Ref	Ref ‖	Ref
Quintile 2	1.47	[0.85, 2.54]	1.24	[0.69, 2.21]	1.44	[0.78, 2.66]
Quintile 3	1.57	[0.91, 2.71]	1.37	[0.77, 2.46]	1.44	[0.77, 2.66]
Quintile 4	2.31**	[1.35, 3.95]	1.76	[0.98, 3.14]	1.66	[0.89, 3.07]
Quintile 5	3.49***	[2.06, 5.92]	3.07***	[1.73, 5.45]	2.94***	[1.60, 5.40]

Biomarker	Model A* OR (95% CI)		Model B† aOR (95% CI)		Model C‡ aOR (95% CI)	
**CRP**						
Quintile 1	Ref ‖	Ref	Ref ‖	Ref	Ref ‖	Ref
Quintile 2	1.42	[0.84, 2.40]	1.42	[0.81, 2.48]	1.42	[0.79, 2.57]
Quintile 3	1.79*	[1.07, 3.01]	1.72	[0.99, 2.99]	1.66	[0.92, 2.98]
Quintile 4	1.90*	[1.14, 3.17]	1.80*	[1.04, 3.11]	1.66	[0.92, 2.99]
Quintile 5	2.12**	[1.26, 3.55]	2.21**	[1.26, 3.86]	2.21*	[1.20, 4.06]
**IL-6**						
Quintile 1	Ref ‖	Ref	Ref ‖	Ref	Ref ‖	Ref
Quintile 2	1.39	[0.81, 2.38]	1.06	[0.60, 1.88]	1.05	[0.58, 1.92]
Quintile 3	2.03**	[1.21, 3.42]	1.76*	[1.01, 3.07]	1.64	[0.90, 3.00]
Quintile 4	2.20**	[1.30, 3.73]	1.84*	[1.04, 3.27]	1.64	[0.88, 3.04]
Quintile 5	2.57***	[1.54, 4.31]	2.01*	[1.14, 3.54]	1.82	[0.99, 3.36]
**sTNF-αR1**						
Quintile 1	Ref ‖	Ref	Ref	Ref	Ref	Ref
Quintile 2	0.65	[0.39, 1.09]	0.56*	[0.32, 0.98]	0.60	[0.33, 1.09]
Quintile 3	0.91	[0.55, 1.50]	0.80	[0.47, 1.36]	0.78	[0.44, 1.39]
Quintile 4	1.30	[0.80, 2.12]	1.07	[0.63, 1.83]	1.08	[0.61, 1.91]
Quintile 5	1.47	[0.90, 2.39]	1.03	[0.60, 1.77]	0.76	[0.42, 1.38]
**sTNF-αR2**						
Quintile 1	Ref ‖	Ref	Ref	Ref	Ref	Ref
Quintile 2	1.37	[0.82, 2.28]	1.38	[0.79, 2.39]	1.44	[0.81, 2.56]
Quintile 3	1.32	[0.79, 2.19]	1.11	[0.65, 1.91]	1.12	[0.63, 1.99]
Quintile 4	1.17	[0.70, 1.97]	0.92	[0.52, 1.61]	0.86	[0.48, 1.57]
Quintile 5	2.06**	[1.24, 3.40]	1.57	[0.90, 2.73]	1.21	[0.66, 2.24]
**Fibrinogen**						
Quintile 1	Ref ‖	Ref	Ref	Ref	Ref	Ref

Biomarker	Model A* OR (95% CI)		Model B† aOR (95% CI)		Model C‡ aOR (95% CI)	
Quintile 2	1.12	[0.67, 1.88]	1.26	[0.72, 2.20]	1.40	[0.77, 2.53]
Quintile 3	1.23	[0.74, 2.05]	1.33	[0.77, 2.30]	1.34	[0.74, 2.41]
Quintile 4	1.37	[0.82, 2.26]	1.26	[0.73, 2.18]	1.31	[0.73, 2.36]
Quintile 5	1.74*	[1.06, 2.87]	1.65	[0.96, 2.86]	1.55	[0.86, 2.80]
**D-dimer**						
Quintile 1	Ref	Ref	Ref	Ref	Ref	Ref
Quintile 2	1.57	[0.96, 2.56]	1.45	[0.86, 2.45]	1.47	[0.84, 2.57]
Quintile 3	1.18	[0.73, 1.92]	0.97	[0.58, 1.62]	0.87	[0.50, 1.51]
Quintile 4	1.28	[0.79, 2.07]	1.08	[0.63, 1.84]	0.98	[0.55, 1.74]
Quintile 5	1.44	[0.90, 2.32]	1.02	[0.61, 1.73]	0.91	[0.52, 1.59]

Abbreviations: sCD163, cluster of differentiation 163; sCD14, cluster of differentiation 14; CCL2, chemokine (C-C motif) ligand 2; ICAM-1, intercellular cell adhesion molecule-1; CRP, C reactive protein; IL-6, interleukin-6; sTNF-αR1, tumor necrosis factor-alpha receptor 1; sTNF-αR2, tumor necrosis factor-alpha receptor 2. Results are presented as odds ratios and 95% CI, $^{*}p < 0.05$, $^{**}p < 0.01$, $^{***}p < 0.001$, || Significant test for trend (p < 0.05).

*Model A: Unadjusted.
†Model B: Adjusted for HIV serostatus, age, race, baseline education, center, cohort.
‡Model C: Adjusted for variables in model B along with cumulative pack years, alcohol consumption since last visit, HCV, BMI, SBP (per 10mm Hg), total cholesterol (per 5mg/dl), HDL (5mg/dl), glucose levels (per 10 mg/dl), and use of medication for hypertension, diabetes and high cholesterol.
