# Supplementary material for: Inflammatory biomarkers and subclinical carotid atherosclerosis in HIV-infected and HIV-uninfected men in the Multicenter AIDS Cohort Study
Source: PLoS One. 2019 Apr 4;14(4):e0214735. doi: 10.1371/journal.pone.0214735 (PMC6448851; doi:10.1371/journal.pone.0214735)
Supplement: S6 Table — (PDF) [file pone.0214735.s007.pdf]

S6 Table. Adjusted associations between inflammatory biomarkers (mutually adjusted for each other) and focal carotid plaque (N=728)

Biomarker	Focal Plaque*	
	aOR (95%CI)	P-value
**sCD163**	2.00 (0.99, 4.07)	0.05
**sCD14**	1.32 (0.64, 2.72)	0.46
**ICAM-1**	1.13 (0.57, 2.25)	0.73
**CCL2**	**3.16 (1.60, 6.22)**	**<0.01**
**CRP**	**2.27 (1.05, 4.90)**	**0.04**
**IL-6**	1.55(0.73, 3.30)	0.25
**sTNF-αR1**	0.54 (0.25, 1.17)	0.12
**sTNF-αR2**	0.76 (0.33, 1.73)	0.51
**Fibrinogen**	0.93 (0.46, 1.88)	0.84
**D-dimer**	0.64 (0.34, 1.20)	0.16

Abbreviations: sCD163, cluster of differentiation 163; sCD14, cluster of differentiation 14; CCL2- chemokine (C-C motif) ligand 2; ICAM-1, intercellular cell adhesion molecule-1; CRP, C reactive protein; IL-6, interleukin-6; sTNF-αR1, tumor necrosis factor-alpha receptor 1; sTNF-αR2, tumor necrosis factor- alpha receptor 2. Results are from a single multivariable model including all ten biomarkers. Results are presented as adjusted odds ratios and 95% CI, bolded results are statistically significant ($p < 0.05$).

*Model adjusted for age, race, baseline education, center, cohort, cumulative pack years, alcohol consumption since last visit, HCV, BMI, SBP (per 10mm Hg), total cholesterol (per 5mg/dl), HDL (5mg/dl), glucose levels (per 10 mg/dl), and use of medication for hypertension, diabetes and high cholesterol.
