# Supplementary material for: Inflammatory biomarkers and subclinical carotid atherosclerosis in HIV-infected and HIV-uninfected men in the Multicenter AIDS Cohort Study
Source: PLoS One. 2019 Apr 4;14(4):e0214735. doi: 10.1371/journal.pone.0214735 (PMC6448851; doi:10.1371/journal.pone.0214735)
Supplement: S7 Table — (PDF) [file pone.0214735.s008.pdf]

S7 Table. Associations between inflammatory biomarkers and focal carotid plaque, by HIV serostatus (n=728)

Biomarker	HIV infected (N=452)						HIV uninfected (N=276)				Interaction term Model C (P-Value)
	Model B*		Model C†		Model D‡		Model B*		Model C†		
	aOR (95% CI)	P-value	aOR (95% CI)	P-value	aOR (95% CI)	P-value	aOR (95% CI)	P-value	aOR (95% CI)	P-value	
**sCD163**	**2.89 [1.39, 6.00]**	**<0.01**	**2.62 [1.15, 5.97]**	**0.02**	**3.39 [1.36, 8.48]**	**0.01**	1.09 [0.39, 3.06]	0.87	1.26 [0.35, 4.57]	0.72	0.29
**sCD14**	1.29 [0.61, 2.72]	0.51	1.00 [0.44, 2.28]	1.00	1.03 [0.43, 2.44]	0.95	3.71 [0.74, 18.59]	0.11	4.91 [0.84, 28.82]	0.08	0.21
**ICAM-1**	1.08 [0.55, 2.12]	0.82	0.77 [0.36, 1.65]	0.50	0.74 [0.33, 1.66]	0.47	**6.48 [2.34, 17.94]**	**<0.001**	**6.8 [2.15, 21.54]**	**<0.01**	**<0.001**
**CCL2**	**4.2 [1.93, 9.11]**	**<0.001**	**3.24 [1.45, 7.24]**	**<0.01**	**3.12 [1.37, 7.10]**	**0.01**	2.12 [0.80, 5.61]	0.13	2.93 [0.98, 8.78]	0.06	0.87
**CRP**	**2.06 [1.03, 4.09]**	**0.04**	2.05 [0.98, 4.28]	0.06	**2.26 [1.05, 4.89]**	**0.04**	2.36 [0.87, 6.35]	0.09	3.23 [0.97, 10.82]	0.06	0.58
**IL-6**	1.86 [0.91, 3.77]	0.09	1.59 [0.73, 3.45]	0.24	1.37 [0.61, 3.07]	0.44	2.35 [0.89, 6.18]	0.08	**3.15 [1.01, 9.81]**	**0.05**	0.34
**sTNF-αR1**	1.19 [0.62, 2.28]	0.61	0.68 [0.33, 1.44]	0.32	0.56 [0.26, 1.21]	0.14	0.59 [0.21, 1.65]	0.31	0.82 [0.25, 2.63]	0.73	0.74
**sTNF-αR2**	**2.03 [1.02, 4.03]**	**0.04**	1.54 [0.72, 3.29]	0.26	1.56 [0.70, 3.51]	0.28	0.73 [0.25, 2.13]	0.57	0.71 [0.22, 2.32]	0.57	0.33
**Fibrinogen**	1.38 [0.72, 2.63]	0.33	1.29 [0.64, 2.60]	0.48	1.36 [0.65, 2.87]	0.42	**3.70 [1.12, 12.23]**	**0.03**	**4.09 [1.10, 15.18]**	**0.04**	0.16
**D-dimer**	0.98 [0.52, 1.84]	0.95	0.92 [0.47, 1.80]	0.80	1.01 [0.50, 2.05]	0.97	1.13 [0.43, 2.98]	0.81	0.76 [0.26, 2.19]	0.61	0.72

Abbreviations: sCD163, cluster of differentiation 163; sCD14, cluster of differentiation 14; CCL2, chemokine (C-C motif) ligand 2; ICAM-1, intercellular cell adhesion molecule-1; CRP, C reactive protein; IL-6, interleukin-6; sTNF-αR1, tumor necrosis factor-alpha receptor 1; sTNF-αR2, tumor necrosis factor-alpha receptor 2. Results presented as adjusted odds ratio (95% CI), bolded results are statistically significant ($p < 0.05$).

*Model B: Adjusted for age, race, baseline education, center, cohort.
†Model C: Adjusted for variables in model B along with cumulative pack years, alcohol consumption since last visit, HCV, BMI, SBP (per 10mm Hg), total cholesterol (per 5mg/dl), HDL (5mg/dl), glucose levels (per 10 mg/dl), and use of medication for hypertension, diabetes and high cholesterol.
‡Model D: Adjusted for variables in Model C along with HIV specific factors, CD4 count, CD4 nadir, diagnosis of AIDS, virologically unsuppressed in the past 5 years.
