# Supplementary material for: Inflammatory biomarkers and subclinical carotid atherosclerosis in HIV-infected and HIV-uninfected men in the Multicenter AIDS Cohort Study
Source: PLoS One. 2019 Apr 4;14(4):e0214735. doi: 10.1371/journal.pone.0214735 (PMC6448851; doi:10.1371/journal.pone.0214735)
Supplement: S8 Table — (PDF) [file pone.0214735.s009.pdf]

S8 Table: Associations between biomarkers and markers of subclinical carotid atherosclerosis among HIV-infected men with suppressed viral load

Biomarker	Plaque (N=372)				cIMT (N=372)				Bifurcation-IMT (N=363)			
	Model C* aOR (95%CI)		Model D† aOR (95%CI)		Model C* ß (95% CI)		Model D† ß (95% CI)		Model C* ß (95% CI)		Model D† ß (95% CI)	
**sCD163**												
Quintile 1	Ref	Ref	Ref	Ref	Ref	Ref	Ref	Ref	Ref	Ref	Ref	Ref
Quintile 2	1.19	[0.49, 2.92]	1.17	[0.46, 2.96]	-0.04	[-0.08, 0.01]	-0.03	[-0.08, 0.02]	0.00	[-0.06, 0.05]	0.01	[-0.05, 0.07]
Quintile 3	1.70	[0.71, 4.08]	1.69	[0.68, 4.22]	0.00	[-0.04, 0.05]	0.00	[-0.04, 0.05]	-0.01	[-0.06, 0.05]	-0.01	[-0.06, 0.05]
Quintile 4	2.08	[0.88, 4.92]	1.96	[0.78, 4.91]	-0.02	[-0.07, 0.02]	-0.01	[-0.06, 0.03]	0.00	[-0.05, 0.06]	0.01	[-0.05, 0.07]
Quintile 5	2.18	[0.88, 5.38]	**2.71**	**[1.01, 7.29]**	-0.03	[-0.08, 0.02]	-0.02	[-0.08, 0.03]	0.00	[-0.06, 0.06]	0.00	[-0.06, 0.06]
**sCD14**												
Quintile 1	Ref	Ref	Ref	Ref	Ref	Ref	Ref	Ref	Ref	Ref	Ref	Ref
Quintile 2	0.49	[0.16, 1.44]	0.45	[0.15, 1.41]	-0.02	[-0.08, 0.04]	-0.03	[-0.08, 0.03]	0.01	[-0.06, 0.08]	0.00	[-0.07, 0.07]
Quintile 3	0.68	[0.26, 1.80]	0.66	[0.25, 1.78]	-0.01	[-0.06, 0.05]	-0.01	[-0.06, 0.05]	0.01	[-0.06, 0.08]	0.01	[-0.06, 0.08]
Quintile 4	0.77	[0.30, 1.98]	0.80	[0.30, 2.09]	0.02	[-0.04, 0.07]	0.01	[-0.05, 0.06]	0.03	[-0.04, 0.09]	0.02	[-0.05, 0.09]
Quintile 5	1.07	[0.41, 2.74]	0.99	[0.38, 2.61]	-0.01	[-0.06, 0.05]	-0.01	[-0.07, 0.05]	0.03	[-0.04, 0.10]	0.03	[-0.04, 0.10]
**ICAM-1**												
Quintile 1	Ref	Ref	Ref	Ref	Ref	Ref	Ref	Ref	Ref	Ref	Ref	Ref
Quintile 2	0.54	[0.22, 1.31]	0.49	[0.20, 1.24]	0.00	[-0.05, 0.05]	0.00	[-0.05, 0.05]	0.05	[-0.01, 0.11]	0.05	[-0.02, 0.11]
Quintile 3	0.51	[0.21, 1.24]	0.45	[0.18, 1.13]	0.00	[-0.05, 0.05]	0.00	[-0.04, 0.05]	0.02	[-0.03, 0.08]	0.03	[-0.03, 0.09]
Quintile 4	**0.30**	**[0.12, 0.72]**	**0.25**	**[0.10, 0.64]**	-0.01	[-0.06, 0.03]	-0.01	[-0.06, 0.04]	0.04	[-0.01, 0.10]	0.05	[-0.02, 0.11]
Quintile 5	0.77	[0.33, 1.81]	0.76	[0.31, 1.85]	0.00	[-0.05, 0.04]	0.00	[-0.05, 0.05]	0.06	[-0.00, 0.12]	0.05	[-0.01, 0.12]
**CCL2**												
Quintile 1	Ref	Ref	Ref	Ref	Ref	Ref	Ref	Ref	Ref	Ref	Ref	Ref
Quintile 2	0.94	[0.35, 2.58]	0.92	[0.33, 2.60]	0.00	[-0.05, 0.05]	0.00	[-0.05, 0.05]	-0.02	[-0.08, 0.04]	-0.01	[-0.08, 0.05]
Quintile 3	2.18	[0.88, 5.40]	2.14	[0.84, 5.47]	0.00	[-0.05, 0.05]	0.00	[-0.05, 0.05]	-0.01	[-0.07, 0.05]	-0.01	[-0.07, 0.05]
Quintile 4	1.88	[0.73, 4.84]	1.85	[0.70, 4.87]	0.00	[-0.05, 0.05]	0.00	[-0.05, 0.05]	-0.01	[-0.07, 0.05]	-0.01	[-0.07, 0.05]
Quintile 5	**3.16**	**[1.27, 7.85]**	**3.15**	**[1.24, 8.01]**	0.00	[-0.04, 0.05]	0.01	[-0.04, 0.06]	0.02	[-0.04, 0.08]	0.02	[-0.04, 0.08]
**CRP**												
Quintile 1	Ref	Ref	Ref	Ref	Ref	Ref	Ref	Ref	Ref	Ref	Ref	Ref

Biomarker	Plaque (N=372)				cIMT (N=372)				Bifurcation-IMT (N=363)			
	Model C* aOR (95%CI)		Model D† aOR (95%CI)		Model C* ß (95% CI)		Model D† ß (95% CI)		Model C* ß (95% CI)		Model D† ß (95% CI)	
Quintile 2	1.14	[0.49, 2.62]	1.14	[0.47, 2.75]	**-0.04**	**[-0.09, -0.00]**	-0.04	[-0.09, 0.00]	**-0.06**	**[-0.12, -0.01]**	**-0.06**	**[-0.12, -0.00]**
Quintile 3	1.41	[0.62, 3.22]	1.41	[0.59, 3.36]	-0.02	[-0.06, 0.03]	-0.01	[-0.06, 0.04]	-0.03	[-0.09, 0.02]	-0.03	[-0.09, 0.03]
Quintile 4	1.15	[0.52, 2.55]	1.05	[0.45, 2.44]	-0.03	[-0.08, 0.01]	-0.03	[-0.07, 0.02]	-0.02	[-0.07, 0.04]	-0.01	[-0.07, 0.04]
Quintile 5	**2.33**	**[1.04, 5.23]**	**2.67**	**[1.15, 6.22]**	0.01	[-0.04, 0.05]	0.01	[-0.03, 0.06]	0.00	[-0.06, 0.05]	0.00	[-0.06, 0.06]
**IL-6**												
Quintile 1	Ref	Ref	Ref	Ref	Ref	Ref	Ref	Ref	Ref	Ref	Ref	Ref
Quintile 2	0.58	[0.23, 1.46]	0.46	[0.18, 1.23]	0.00	[-0.05, 0.04]	0.00	[-0.05, 0.04]	0.02	[-0.03, 0.08]	0.02	[-0.04, 0.08]
Quintile 3	**2.84**	**[1.22, 6.58]**	**2.51**	**[1.07, 5.88]**	-0.02	[-0.06, 0.03]	-0.01	[-0.06, 0.03]	0.03	[-0.02, 0.08]	0.03	[-0.02, 0.09]
Quintile 4	2.13	[0.90, 5.04]	1.75	[0.73, 4.17]	0.02	[-0.02, 0.07]	0.03	[-0.01, 0.08]	**0.06**	**[0.00, 0.12]**	**0.07**	**[0.01, 0.12]**
Quintile 5	1.89	[0.78, 4.59]	1.76	[0.71, 4.34]	0.02	[-0.02, 0.07]	0.03	[-0.02, 0.07]	0.03	[-0.02, 0.09]	0.03	[-0.03, 0.09]
**sTNF-αR1**												
Quintile 1	Ref	Ref	Ref	Ref	Ref	Ref	Ref	Ref	Ref	Ref	Ref	Ref
Quintile 2	**0.35**	**[0.15, 0.82]**	**0.33**	**[0.14, 0.81]**	0.00	[-0.04, 0.05]	0.00	[-0.05, 0.05]	-0.02	[-0.07, 0.04]	-0.02	[-0.08, 0.04]
Quintile 3	0.56	[0.25, 1.24]	0.58	[0.26, 1.32]	-0.01	[-0.05, 0.04]	-0.01	[-0.05, 0.04]	-0.02	[-0.08, 0.03]	-0.02	[-0.08, 0.03]
Quintile 4	0.50	[0.22, 1.14]	**0.42**	**[0.18, 0.98]**	0.00	[-0.05, 0.04]	-0.01	[-0.05, 0.04]	0.00	[-0.06, 0.05]	-0.01	[-0.06, 0.05]
Quintile 5	0.56	[0.24, 1.31]	0.54	[0.23, 1.27]	0.01	[-0.04, 0.06]	0.01	[-0.04, 0.06]	0.02	[-0.04, 0.08]	0.02	[-0.04, 0.08]
**sTNF-αR2**												
Quintile 1	Ref	Ref	Ref	Ref	Ref	Ref	Ref	Ref	Ref	Ref	Ref	Ref
Quintile 2	1.16	[0.52, 2.60]	1.27	[0.54, 2.98]	0.01	[-0.03, 0.06]	0.02	[-0.03, 0.07]	-0.02	[-0.08, 0.03]	-0.02	[-0.08, 0.04]
Quintile 3	1.30	[0.58, 2.92]	1.36	[0.58, 3.21]	0.00	[-0.05, 0.04]	0.01	[-0.04, 0.05]	0.01	[-0.05, 0.06]	0.02	[-0.04, 0.08]
Quintile 4	0.86	[0.38, 1.94]	0.88	[0.38, 2.06]	0.01	[-0.04, 0.05]	0.02	[-0.03, 0.06]	0.00	[-0.05, 0.06]	0.01	[-0.05, 0.07]
Quintile 5	1.61	[0.69, 3.75]	1.60	[0.67, 3.84]	0.00	[-0.04, 0.05]	0.01	[-0.04, 0.06]	0.00	[-0.06, 0.06]	0.00	[-0.06, 0.07]
**Fibrinogen**												
Quintile 1	Ref	Ref	Ref	Ref	Ref	Ref	Ref	Ref	Ref	Ref	Ref	Ref
Quintile 2	0.63	[0.28, 1.41]	0.66	[0.29, 1.50]	0.01	[-0.03, 0.05]	0.02	[-0.03, 0.06]	0.01	[-0.04, 0.07]	0.01	[-0.04, 0.07]
Quintile 3	1.65	[0.77, 3.53]	1.77	[0.81, 3.87]	0.02	[-0.02, 0.07]	0.03	[-0.01, 0.07]	0.00	[-0.05, 0.06]	0.01	[-0.05, 0.06]
Quintile 4	0.91	[0.42, 1.97]	0.99	[0.44, 2.18]	0.01	[-0.03, 0.05]	0.02	[-0.03, 0.06]	0.02	[-0.03, 0.07]	0.02	[-0.03, 0.08]
Quintile 5	1.42	[0.65, 3.11]	1.51	[0.67, 3.38]	0.04	[-0.01, 0.08]	**0.05**	**[0.00, 0.09]**	0.02	[-0.04, 0.07]	0.02	[-0.04, 0.08]
**D-dimer**												

Biomarker	Plaque (N=372)				cIMT (N=372)				Bifurcation-IMT (N=363)			
	Model C* aOR (95%CI)		Model D† aOR (95%CI)		Model C* ß (95% CI)		Model D† ß (95% CI)		Model C* ß (95% CI)		Model D† ß (95% CI)	
Quintile 1	Ref	Ref	Ref	Ref	Ref	Ref	Ref	Ref	Ref	Ref	Ref	Ref
Quintile 2	1.72	[0.84, 3.54]	1.81	[0.85, 3.82]	-0.03	[-0.07, 0.01]	-0.03	[-0.07, 0.01]	-0.01	[-0.06, 0.04]	0.00	[-0.05, 0.05]
Quintile 3	0.89	[0.42, 1.90]	0.89	[0.41, 1.93]	-0.04	[-0.08, 0.00]	-0.04	[-0.08, 0.01]	-0.02	[-0.08, 0.03]	-0.02	[-0.07,0.03]
Quintile 4	0.81	[0.35, 1.89]	0.76	[0.32, 1.81]	-0.03	[-0.08, 0.01]	-0.03	[-0.08, 0.02]	-0.02	[-0.08, 0.03]	-0.02	[-0.07, 0.04]
Quintile 5	1.02	[0.47, 2.19]	1.09	[0.49, 2.42]	-0.04	[-0.08, 0.00]	-0.04	[-0.08, 0.00]	-0.04	[-0.09, 0.01]	-0.04	[-0.09, 0.02]

Abbreviations: sCD163, cluster of differentiation 163; sCD14, cluster of differentiation 14; CCL2, chemokine (C-C motif) ligand 2; ICAM-1, intercellular cell adhesion molecule-1; CRP, C reactive protein; IL-6, interleukin-6; sTNF-αR1, tumor necrosis factor-alpha receptor 1; sTNF-αR2, tumor necrosis factor-alpha receptor 2. Results presented as adjusted odds ratios (95% CI), bolded results are statistically significant ($p < 0.05$).

*Model C: Adjusted for age, race, baseline education, center, cohort, cumulative pack years, alcohol consumption since last visit, HCV, BMI, SBP (per 10mm Hg), total cholesterol (per 5mg/dl), HDL (5mg/dl), glucose levels (per 10 mg/dl), and use of medication for hypertension, diabetes and high cholesterol.

†Model D: Adjusted for variables in Model C along with HIV specific factors, CD4 count, CD4 nadir, diagnosis of AIDS.
