# Supplementary material for: Inflammatory biomarkers and subclinical carotid atherosclerosis in HIV-infected and HIV-uninfected men in the Multicenter AIDS Cohort Study
Source: PLoS One. 2019 Apr 4;14(4):e0214735. doi: 10.1371/journal.pone.0214735 (PMC6448851; doi:10.1371/journal.pone.0214735)
Supplement: S10 Table — (PDF) [file pone.0214735.s011.pdf]

S10 Table. Associations between the fifth quintile of the inflammatory biomarkers and intima media thickness at the right common carotid artery, by HIV serostatus (n=728)

Biomarkers	HIV-infected (n=452)				HIV-uninfected (n=276)				Interaction Model (p value)
	Model B* ß (95% CI)		Model C† ß (95% CI)		Model B* ß (95% CI)		Model C† ß (95% CI)		
**sCD163**	-0.01	[-0.05, 0.03]	-0.01	[-0.05, 0.03]	0.05	[-0.00, 0.11]	0.02	[-0.03, 0.08]	0.15
**sCD14**	0.00	[-0.05, 0.04]	0.00	[-0.04, 0.05]	-0.01	[-0.10, 0.08]	-0.03	[-0.12, 0.06]	0.88
**ICAM-1**	-0.01	[-0.05, 0.04]	-0.01	[-0.05, 0.04]	0.06*	[0.01, 0.11]	0.03	[-0.02, 0.08]	0.25
**CCL2**	0.01	[-0.03, 0.06]	0.00	[-0.04, 0.04]	0.01	[-0.05, 0.06]	0.00	[-0.05, 0.05]	0.68
**CRP**	0.01	[-0.03, 0.05]	0.00	[-0.04, 0.04]	0.07**	[0.02, 0.11]	0.04	[-0.01, 0.10]	0.23
**IL-6**	0.03	[-0.02, 0.07]	0.01	[-0.03, 0.05]	0.02	[-0.03, 0.07]	-0.01	[-0.06, 0.04]	0.87
**sTNF-αR1**	0.04	[-0.00, 0.08]	0.02	[-0.02, 0.07]	0.01	[-0.04, 0.06]	0.01	[-0.04, 0.06]	0.73
**sTNF-αR2**	0.02	[-0.02, 0.06]	0.02	[-0.02, 0.06]	0.07**	[0.02, 0.13]	0.07*	[0.01, 0.12]	0.12
**Fibrinogen**	0.04*	[0.00, 0.08]	0.03	[-0.01, 0.07]	0.09***	[0.04, 0.14]	0.07**	[0.02, 0.12]	0.29
**D-dimer**	-0.04	[-0.07, 0.00]	-0.03	[-0.07, 0.00]	-0.03	[-0.08, 0.02]	-0.04	[-0.08, 0.01]	0.84

Abbreviations: sCD163, cluster of differentiation 163; sCD14, cluster of differentiation 14; CCL2, chemokine (C-C motif) ligand 2; ICAM-1, intercellular cell adhesion molecule-1; CRP, C reactive protein; IL-6, interleukin-6; sTNF-αR1, tumor necrosis factor-alpha receptor 1; sTNF-αR2, tumor necrosis factor-alpha receptor 2. Results presented as ß coefficients and 95% confidence intervals, * $p < 0.05$, ** $p < 0.01$, *** $p < 0.001$

*Model B: Adjusted for age, race, baseline education, center, cohort.
†Model C: Adjusted for variables in model B along with cumulative pack years, alcohol consumption since last visit, HCV, BMI, SBP (per 10mm Hg), total cholesterol (per 5mg/dl), HDL (5mg/dl), glucose levels (per 10 mg/dl), and use of medication for hypertension, diabetes and high cholesterol.
