# Supplementary material for: Inflammatory biomarkers and subclinical carotid atherosclerosis in HIV-infected and HIV-uninfected men in the Multicenter AIDS Cohort Study
Source: PLoS One. 2019 Apr 4;14(4):e0214735. doi: 10.1371/journal.pone.0214735 (PMC6448851; doi:10.1371/journal.pone.0214735)
Supplement: S11 Table — (PDF) [file pone.0214735.s012.pdf]

S11 Table. Associations between inflammatory biomarkers and IMT at the bifurcation of the right common carotid artery (N=672)

Biomarker	Model A* ß (95% CI)		Model B† ß (95% CI)		Model C‡ ß (95% CI)	
**sCD163**						
Quintile 1	Ref	Ref	Ref	Ref	Ref	Ref
Quintile 2	0.02	[-0.02, 0.06]	0.01	[-0.02, 0.05]	0.02	[-0.02, 0.05]
Quintile 3	0.02	[-0.02, 0.06]	0.02	[-0.02, 0.06]	0.02	[-0.01, 0.06]
Quintile 4	0.03	[-0.01, 0.07]	0.03	[-0.01, 0.07]	0.03	[-0.01, 0.07]
Quintile 5	0.03	[-0.01, 0.07]	0.02	[-0.02, 0.06]	0.02	[-0.02, 0.07]
**sCD14**						
Quintile 1	Ref	Ref	Ref	Ref	Ref	Ref
Quintile 2	-0.01	[-0.05, 0.03]	-0.01	[-0.05, 0.03]	-0.01	[-0.05, 0.03]
Quintile 3	0.01	[-0.03, 0.05]	0.01	[-0.03, 0.05]	0.01	[-0.03, 0.04]
Quintile 4	0.00	[-0.04, 0.04]	0.01	[-0.03, 0.05]	0.02	[-0.02, 0.06]
Quintile 5	0.01	[-0.03, 0.05]	0.01	[-0.04, 0.05]	0.01	[-0.03, 0.05]
**ICAM-1**						
Quintile 1	Ref	Ref	Ref	Ref	Ref	Ref
Quintile 2	0.02	[-0.02, 0.06]	0.03	[-0.01, 0.07]	0.03	[-0.01, 0.06]
Quintile 3	0.02	[-0.02, 0.06]	0.03	[-0.01, 0.07]	0.03	[-0.00, 0.07]
Quintile 4	0.03	[-0.01, 0.07]	0.03	[-0.01, 0.07]	0.02	[-0.02, 0.06]
Quintile 5	0.05**	[0.01, 0.09]	0.06**	[0.02, 0.10]	0.05*	[0.01, 0.09]
**CCL2**						
Quintile 1	Ref	Ref	Ref	Ref	Ref	Ref
Quintile 2	0.00	[-0.04, 0.04]	0.00	[-0.03, 0.04]	0.01	[-0.03, 0.05]
Quintile 3	0.01	[-0.03, 0.05]	0.01	[-0.03, 0.05]	0.01	[-0.03, 0.05]
Quintile 4	0.03	[-0.01, 0.07]	0.01	[-0.03, 0.05]	0.01	[-0.03, 0.05]
Quintile 5	0.06**	[0.02, 0.10]	0.05*	[0.01, 0.09]	0.04*	[0.00, 0.08]
**CRP**						
Quintile 1	Ref	Ref	Ref	Ref	Ref	Ref
Quintile 2	-0.03	[-0.07, 0.01]	-0.03	[-0.06, 0.01]	-0.03	[-0.07, 0.00]
Quintile 3	0.01	[-0.03, 0.04]	0	[-0.03, 0.04]	-0.01	[-0.05, 0.03]
Quintile 4	0.03	[-0.00, 0.07]	0.03	[-0.00, 0.07]	0.02	[-0.02, 0.05]
Quintile 5	0.04*	[0.00, 0.08]	0.03	[-0.01, 0.07]	0.02	[-0.02, 0.05]
**IL-6**						
Quintile 1	Ref	Ref	Ref	Ref	Ref	Ref
Quintile 2	0.04*	[0.00, 0.08]	0.03	[-0.01, 0.06]	0.02	[-0.02, 0.06]
Quintile 3	0.04*	[0.00, 0.08]	0.02	[-0.01, 0.06]	0.01	[-0.03, 0.05]
Quintile 4	0.08***	[0.04, 0.12]	0.06**	[0.02, 0.10]	0.05*	[0.01, 0.09]

Biomarker	Model A* ß (95% CI)		Model B† ß (95% CI)		Model C‡ ß (95% CI)	
Quintile 5	0.06**	[0.02, 0.09]	0.03	[-0.01, 0.07]	0.02	[-0.02, 0.06]
**sTNF-αR1**						
Quintile 1	Ref	Ref	Ref	Ref	Ref	Ref
Quintile 2	0.02	[-0.02, 0.06]	0.02	[-0.01, 0.06]	0.03	[-0.01, 0.07]
Quintile 3	0.02	[-0.02, 0.06]	0.02	[-0.02, 0.06]	0.02	[-0.02, 0.06]
Quintile 4	0.01	[-0.02, 0.05]	0.01	[-0.03, 0.05]	0.02	[-0.02, 0.06]
Quintile 5	0.05**	[0.01, 0.09]	0.04	[-0.00, 0.08]	0.04*	[0.00, 0.08]
**sTNF-αR2**						
Quintile 1	Ref	Ref	Ref	Ref	Ref	Ref
Quintile 2	0.01	[-0.03, 0.05]	0.00	[-0.04, 0.04]	0.00	[-0.04, 0.04]
Quintile 3	0.01	[-0.02, 0.05]	0.01	[-0.03, 0.04]	0.01	[-0.03, 0.05]
Quintile 4	-0.01	[-0.05, 0.03]	-0.01	[-0.05, 0.03]	-0.01	[-0.05, 0.03]
Quintile 5	0.03	[-0.01, 0.07]	0.01	[-0.03, 0.05]	0.02	[-0.02, 0.06]
**Fibrinogen**						
Quintile 1	Ref	Ref	Ref	Ref	Ref	Ref
Quintile 2	0.00	[-0.04, 0.04]	0.01	[-0.03, 0.04]	0.00	[-0.03, 0.04]
Quintile 3	0.02	[-0.02, 0.06]	0.00	[-0.03, 0.04]	0.00	[-0.04, 0.03]
Quintile 4	0.03	[-0.01, 0.07]	0.01	[-0.02, 0.05]	0.00	[-0.04, 0.04]
Quintile 5	0.07***	[0.03, 0.11]	0.05*	[0.01, 0.09]	0.03	[-0.01, 0.07]
**D-dimer**						
Quintile 1	Ref	Ref	Ref	Ref	Ref	Ref
Quintile 2	0.02	[-0.02, 0.06]	0.01	[-0.03, 0.05]	0.01	[-0.02, 0.05]
Quintile 3	0.01	[-0.03, 0.05]	-0.01	[-0.05, 0.02]	-0.01	[-0.05, 0.02]
Quintile 4	0.05*	[0.01, 0.09]	0.02	[-0.02, 0.06]	0.02	[-0.01, 0.06]
Quintile 5	0.02	[-0.02, 0.06]	-0.02	[-0.06, 0.01]	-0.02	[-0.06, 0.02]

Abbreviations: sCD163, cluster of differentiation 163; sCD14, cluster of differentiation 14; CCL2, chemokine (C-C motif) ligand 2; ICAM-1, intercellular cell adhesion molecule-1; CRP, C reactive protein; IL-6, interleukin-6; sTNF-αR1, tumor necrosis factor-alpha receptor 1; sTNF-αR 2, tumor necrosis factor-alpha receptor 2. Results presented as ß coefficients and 95% confidence intervals, * $p < 0.05$, ** $p < 0.01$, *** $p < 0.001$

*Model A: Unadjusted.
†Model B: Adjusted for HIV serostatus, age, race, baseline education, center, cohort.
‡Model C: Adjusted for variables in model B along with cumulative pack years, alcohol consumption since last visit, HCV, BMI, SBP (per 10mm Hg), total cholesterol (per 5mg/dl), HDL (5mg/dl), glucose levels (per 10 mg/dl), and use of medication for hypertension, diabetes and high cholesterol.
